# Boundary Layer Separation and Reattachment Detection on Airfoils by Thermal Flow Sensors

**DOI:** 10.3390/s121114292

**Published:** 2012-10-24

**Authors:** Hannes Sturm, Gerrit Dumstorff, Peter Busche, Dieter Westermann, Walter Lang

**Affiliations:** 1 IMSAS (Institute for Microsensors, -actuators and -systems), MCB (Microsystems Center Bremen), University of Bremen, Otto-Hahn-Allee, Bldg. NW1, 28359 Bremen, Germany; E-Mails: hsturm@imsas.uni-bremen.de (H.S.); gdumstorff@imsas.uni-bremen.de (G.D.); 2 Deutsche Wind Guard Wind Tunnel Services GmbH, Oldenburger Straβe 65, 26316 Varel, Germany; E-Mails: p.busche@windguard.de (P.B.); d.westermann@windguard.de (D.W.)

**Keywords:** boundary layer separation, flow separation, stall, reattachment, airfoil, wind tunnel, thermal flow sensor, flexible, polyimide, thermopile

## Abstract

A sensor concept for detection of boundary layer separation (flow separation, stall) and reattachment on airfoils is introduced in this paper. Boundary layer separation and reattachment are phenomena of fluid mechanics showing characteristics of extinction and even inversion of the flow velocity on an overflowed surface. The flow sensor used in this work is able to measure the flow velocity in terms of direction and quantity at the sensor's position and expected to determine those specific flow conditions. Therefore, an array of thermal flow sensors has been integrated (flush-mounted) on an airfoil and placed in a wind tunnel for measurement. Sensor signals have been recorded at different wind speeds and angles of attack for different positions on the airfoil. The sensors used here are based on the change of temperature distribution on a membrane (calorimetric principle). Thermopiles are used as temperature sensors in this approach offering a baseline free sensor signal, which is favorable for measurements at zero flow. Measurement results show clear separation points (zero flow) and even negative flow values (back flow) for all sensor positions. In addition to standard silicon-based flow sensors, a polymer-based flexible approach has been tested showing similar results.

## 1. Introduction

Boundary layer separation (also flow separation, stall) on overflowed surfaces is a common effect in fluid mechanics. Due to its unstable flow profile combined with drag increase, high energy loss and—in case of airfoils—reduced lift, this effect is mostly unwanted for most applications and even dangerous in aviation [1,2]. The use of shear stress sensors for online detection and even prevention of flow separation could give a better understanding of this still incompletely understood effect.

### 1.1. Theory on Flow Separation

**Figure 1.**
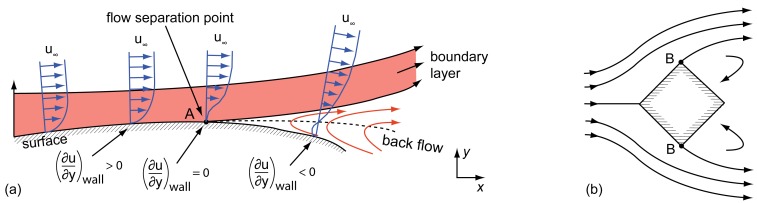
Pressure-based (**a**) and geometrically-based (**b**) flow separation [2,3]. The separation points are marked with A and B. Reproduced with permission from Vogel Buchverlag [3]

When flow separation occurs on a certain surface, the velocity boundary layer becomes detached from it, leading to unsteady flow conditions [2–4]. The phenomenon can basically be explained by the pressure conditions existing on the surface [4]: Figure 1(a) shows an airfoil within a certain flow. When a fluid particle reaches the airfoil's profile at the front (side facing the flow), it will be redirected and therefore accelerated up to a maximum velocity. From that velocity peak down to the trailing edge, the fluid is then decelerated again due to an increasing static pressure (region of adverse pressure gradient)—a behavior also known as pressure recovery or diffusion [5]. When assuming an inviscid flow—this can only be done at an adequate distance from the airfoil and its boundary layers—the pressure conditions can be approximated by *Bernoulli*'s equation [6]:

(1) $$\vec{\nabla }\left(p_{\text{stat}}+\frac{\rho }{2}u^{2}\right)=0$$

where *p*_stat_ is the static pressure, $\frac{\rho }{2}u^{2}$ the dynamic pressure and ∇⃗ the *Nabla*-operator $\vec{\nabla }=\left(\frac{\partial }{\partial x},\frac{\partial }{\partial y},\frac{\partial }{\partial z}\right)$. Considering the airfoil's profile in combination with Equation (1), it is assumed that a particle passing the airfoil has a constant total energy undergoing a transformation from high static (pressure) energy into high kinetic energy and back to static (pressure) energy again. The velocity of the particle in flow direction is thereby, of course, correlated to the kinetic energy leading to a low velocity at the front, a maximum velocity in the middle and a low velocity again at the airfoil's rear (side not facing the flow) [1–3].

When considering friction within the model, a certain amount of the fluid particles' total energy is lost leading to a lower kinetic energy. Thus, at certain flow conditions, there will be not enough kinetic energy of the fluid particles to counteract the increasing static pressure at the airfoil's rear. Especially fluid particles in the boundary layer suffering from the highest amount of friction will reduce their velocity to zero and start to follow a pressure gradient directed to the outer flow field. This pressure distribution leads to a movement in *y*-direction and even flow directed backwards to the overall flow direction [7,8].

Figure 1 illustrates two types of flow separation. However, both types can be led back to the same explanation above. The pressure based flow separation, shown in Figure 1(a), is caused by an adverse pressure gradient occurring at profiles without sharp edges (e.g., airfoil). In case of geometrical aberration (e.g., house wall), as shown in Figure 1(b), a flow separation is called geometrically-based [1,3].

A mathematical explanation of the flow separation background for the stationary case can be given by the *Navier*–*Stokes* equation [6]:

(2) $$\left(\vec{v}\cdot \vec{\nabla }\right)\cdot \vec{v}+\frac{\partial \vec{v}}{\partial t}=\frac{1}{\rho }\vec{F}-\frac{1}{\rho }\vec{\nabla }p+\nu \Delta \vec{v}$$

with *v⃗* = (*u*, *v*, *w*) as the field vector of velocity, *ρ* as density and *ν* as kinematic viscosity. *F⃗* describes the amount of the external force per volume (force density per volume) and ∇*_p_* the amount of the pressure force per volume in ^N^/m^3^. *ν*Δ*v⃗* represents the influence of friction within the equation where $\Delta =\left(\frac{\partial^{2}}{\partial x^{2}}+\frac{\partial^{2}}{\partial y^{2}}+\frac{\partial^{2}}{\partial z^{2}}\right)$ is the *Laplace* operator [6].

For the following statements, all variables do not depend on the third coordinate *z.* Thus, *z* and *w* will further be neglected. If we now consider a position at the wall with *y* = 0, the definitions of the boundary layer give us a simplification with *y* = 0 and *v⃗* = 0 (*u* = 0, *v* = 0). In a stationary case with $\frac{\partial \vec{v}}{\partial t}=0$, which is free of external forces with *F⃗* = 0, Equation (2) can further be simplified to [7,9]:

(3) $$0=-\frac{1}{\rho }\frac{\partial p}{\partial x}+\nu \frac{\partial^{2}u}{\partial y^{2}}.$$

according to Equation (3), there are two important cases with $\frac{\partial p}{\partial x}<0$ and $\frac{\partial p}{\partial x}>0$, which can be distinguished here. The resulting flow profiles are illustrated in Figure 3.

**Figure 2.**
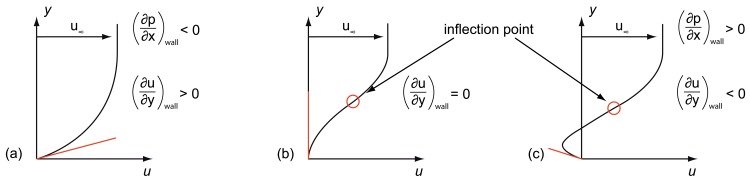
Flow profile on airfoil before (**a**), while (**b**) and after (**c**) boundary layer separation according to Christy and Genc *et al.*[8,9].

The first case with $\frac{\partial p}{\partial x}<0$ results in $\frac{\partial^{2}u}{\partial y^{2}}<0$giving a negative curvature for *u* (*y*), meaning the slope of the function *u* (*y*) decreases for all values of *y.* Due to the fact that *u* (*y*) always tends to a positive value of *u*_∞_, a trend of the function can be given, as illustrated in Figure 2(a): The figure shows the typical velocity distribution over *y* within a laminar boundary layer of an overflowed surface starting at *u*(*0*) = 0 to *u* (*y ≫* 0) = *u*_∞_[7–9]. In the second case the adverse pressure distribution $\frac{\partial p}{\partial x}>0$ results in a positive curvature with $\frac{\partial^{2}u}{\partial y^{2}}>0$, meaning the slope of the function *u* (*y*) has to increase first. Again at the end of the boundary layer a finite velocity of *u_∞_* has to be reached corresponding to a slope $\frac{\partial u}{\partial y}=0$. Therefore, the slope has to have a maximum value before it decreases again. The maximum value of the slope is also the inflection point of the function *u*(*y*). A further decrease of $\frac{\partial p}{\partial x}$allows the possibility of [7,9]:

(4) $${\frac{\partial u}{\partial y}|}_{\text{wall}}=0,$$

which indicates the point of flow separation, as shown in Figure 2(b). Even negative values are possible, as shown in Figure 2(c), indicating back flow after flow separation [7,9].

The results from the Navier–Stokes equation showed the possibility of a flow separation on an overflowed surface. It will now be analyzed whether the phenomenon of reattachment of the boundary layer can be interpreted in the same way.

### 1.2. Reattachment and Separation Bubble Formation at Low Reynolds Numbers

When flow separation occurs, the presence of an either laminar or turbulent flow condition will be essential for the developed flow profile, especially the presence of a flow reattachment and separation bubble formation. According to *Schlichting*, the current state of the flow conditions can be calculated by the *Reynolds* number [2]. A critical value of

(5) $${\text{Re}}_{\text{crit}}=\frac{u_{\infty }l}{\nu }\approx 5\cdot 10^{5},$$

with *l* as characteristic length shows the approximated transition between laminar (< 5 × 10^5^) and turbulent (> 5 × 10^5^) flow conditions of an overflowed flat plate. A precise value cannot be given here, since the real flow conditions depend on the airfoil's shape and roughness, pressure distribution and further factors [7,10,11].

At *Reynolds* numbers of 10^4^ or lower—implicating clear laminar flow conditions on an overflowed flat plate—a separated flow shows reattachment under certain circumstances (adverse pressure gradient) only. Having those laminar conditions (meaning we expect a laminar boundary layer), flow separation usually occurs instantly since laminar boundary layers can handle only small adverse pressure gradients without separation [7,8]. When assuming *Reynolds* numbers of 10^6^ or higher—implicating clear turbulent flow conditions on an overflowed flat plate—the characteristics of the separation are different to the laminar ones. Here, separation usually occurs with very high angles from the trailing edge moving forward with increasing incidence [7,8].

**Figure 3.**
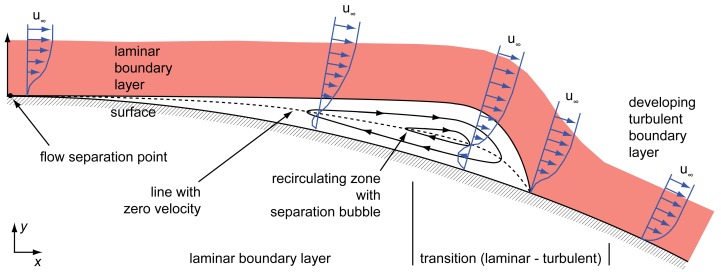
Separation bubble due to reattachment of boundary layer occurring at low *Reynolds* numbers according to Gad-el-Hak and Genc *et al*., [7,8]. It should be mentioned here that the size of the boundary layer in *y*-direction is usually much smaller than the size of the separation bubble.

Other flow conditions show up for *Reynolds* numbers in the range between 10^4^ and 10^6^. Here, a separated flow also occurs, which is continuing along a tangent of the surface at the point of flow separation, as shown in Figure 3. The separated flow descends into a transition from laminar to turbulent flow forming a separation bubble. This phenomenon occurs often at those conditions and can be led back to the increasing distance of the boundary layer to the surface, which results in a higher transition susceptibility [7,8]. According to Gad-el-Hak, turbulent entrainment forces the flow back to the surface, causing the mentioned separation bubble [7].

Figure 3 illustrates a typical airfoil setup with an occurring flow separation followed by a reattachment. The figure shows the velocity profile on an airfoil's surface with the recirculating zone inside the separation bubble. As it can be concluded from Figure 3, a measurement of the actual flow at the separation point and point of reattachment should be possible by a measurement of zero flow in *x*-direction for *y* close to the wall. Due to Equation (4), this condition is fulfilled close to the separation and reattachment point only.

In the following, a measurement setup is presented quantifying the actual mean flow profile in *x* direction for *y* close to the wall, giving us the chance to determine either the point of separation or reattachment.

## 2. Thermal Flow Sensors for Flow Separation Detection—A State of the Art

The exact position of flow separation, which shows the limit of the angle of attack (AOA) prior to decay of the lift coefficient and the corresponding increase of the drag coefficient, is of great interest in many fields like wind power plants and turbines. Especially in aviation, flow separation has to be strongly valuated due to its high risks of losing controllability.

There are already approaches of thermal flow sensors for flow separation detection. *Liu et al*. presents a shear stress sensor based on the anemometric principle: A hot wire on a polyimide foil is heated up by a constant electrical current and the voltage measured giving information about the current temperature of the wire. The occurrence of a fluid flow on the sensor will reduce the temperature, which can be seen as change of voltage of the sensor [12]. In [13] a hot wire (here a polysilicon heater on a silicon nitride membrane) is used as well. Similar state-of-the-art hot film sensors for flow and heat transfer measurement have been presented by Guo *et al*. [14] and Spencer *et al*. [15]. Another approach has been made by Buder *et al*. [16]. Here, two nickel hot wires have been placed on a polyimide substrate with a cavity. The convective heat transfer between both hot wires is measured, a basic concept also known as calorimetric principle. In this work, a calorimetric measurement principle is used as well. However, for the temperature measurement, the thermoelectric effect is exploited. This approach is expected to give different results with regard to accuracy and reproducibility on flow separation measurements than the approaches used before. Benefits to be pointed out are their baseline free measurement results: An output signal of zero will represent a real zero flow combined with a highest sensitivity at this point. An exact description about the used sensors (the non-flexible approach is state-of-the-art) is given next.

## 3. Sensor Setup

### 3.1. Thermal Flow Sensors Based on Calorimetric Principle

**Figure 4.**
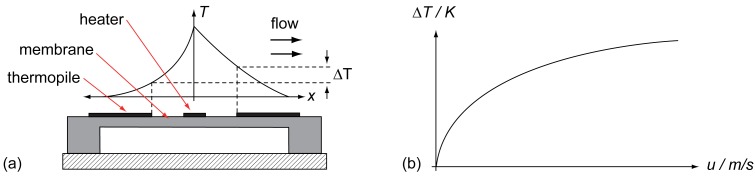
Cross section (schematic) of (**a**) a thermal flow sensor and (**b**) its characteristic line [19].

The thermal flow sensor consists of a heater and thermoelectric temperature sensors symmetrical to both sides of the heater. For thermal isolation, all parts are placed on a membrane. In operating mode, the heater will be heated up giving thermal energy to its surrounding, which is detected by both temperature sensors in the same way. In case of zero flow, the temperature distribution on the membrane will be symmetric, leading to the same detected temperature and therefore to no temperature difference between both thermopiles [17,18]. A presence of a fluid flow over the membrane lateral to the heater—as depicted in Figure 4(a)—causes an asymmetric temperature distribution leading to a measurable temperature difference between both temperature sensors. So, the temperature difference can be interpreted as a flow [18,19].

There are different ways of operating the heater as well as different kinds of temperature sensors possible. In most cases, a constant current (CC) or constant power (CP) is used as operational mode for the heater due to its simplicity. A constant temperature mode (CT) is also used by controlling the heater's temperature-dependent electrical resistance. The effort of realizing a CT mode is higher but it comes along with a higher detectable flow range of the sensor [19,20]. The temperature sensors can be resistive or based on the thermoelectric effect (thermopiles) [21]. When using thermopiles, the thermoelectric effect is converting a temperature difference Δ*T* directly into a voltage

(6) $$U_{\text{AB}}=\alpha_{\text{AB}}\cdot \Delta T,$$

where α_AB_ represents the relative *Seebeck* coefficient of the used thermopile materials *A* and *B.* More information about this topic can be found in [21]. Figure 4(b) shows the characteristic line of a thermal flow sensor in combination with a thermopile-based measurement. As already mentioned, the temperature difference is used for measurement demonstrating the differential principle of the sensor. The principle comes along with a reduction of cross sensitivities and therefore a higher reproducibility of the sensor signal. Furthermore, the output signal of a thermopile-based measurement is baseline free when compared with state-of-the-art resistive approaches on boundary layer detection [12,13,16,22]. Therefore, this approach has been chosen as basic measurement principle for the measurements presented further down.

### 3.2. Sensor Fabrication and Airfoil Integration

As already mentioned, the flow sensor used in this work consists of a combination of heater and thermopiles placed on a membrane [21]. The thermopiles consist of an *in-situ* doped polysilicon and an alloy of 90% tungsten and 10% titanium (WTi). For the heater the WTi alloy has also been used. Both functional elements are embedded in a 600 nm thin membrane made of silicon-rich low-stress LPCVD (low pressure chemical vapor deposition) silicon nitride (SiN). The membrane material deposition with its low tensile stress of +200 MPa has been designed for achieving a high mechanical stability only. A high chemical and thermal stability is also given since the SiN membrane deposition on the functional layers (passivation) is done as LPCVD process at 800 °C. This could be achieved due to diffusion barriers of titanium nitride (TiN) between the polysilicon and WTi used in the thermopile process. More detailed information about the whole process concerning the non-flexible flow sensor can be found in Buchner *et al*. [21]. For the flexible approach, the 380 *μ*m silicon substrate is replaced by a 18 *μ*m thick polyimide. The whole transfer process is shown in [23,24].

**Figure 5.**
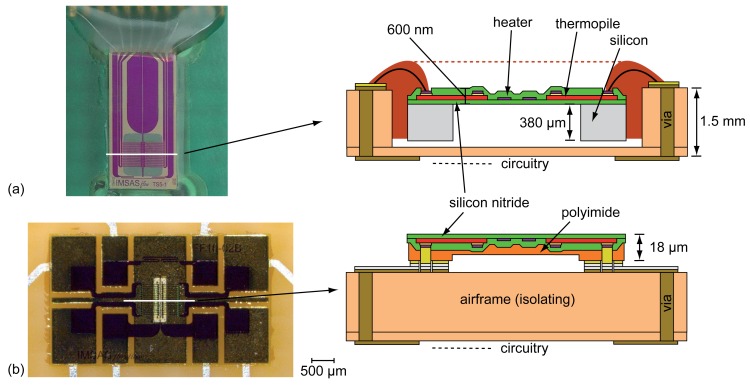
Basic approaches of integrating (**a**) non-flexible and (**b**) flexible thermal flow sensors on airfoils. For the schematics, a cross-sectional area is shown.

Due to its high importance for the aviation sector, an integration of thermal flow sensors has been done on airfoils. As it can be seen in Figure 5, there are two basic concepts of flow sensors used in the following experimental setup.

Figure 5(a) shows an integrated silicon based thermal flow sensor and a schematic of its cross-sectional view. Except for the bond wires (electronic contacting), the whole sensor has been flush-mounted to avoid turbulences on steps that could disturb the measurements. The electronic contacting by bond wires is—due to its height—placed far away from the measuring part of the sensor. As it can be seen, a flush mounting of a silicon-based thermal flow sensor needs another cavity for placing the sensor in addition to the vias for electric contacting, which always affects the structural health of the wing. Therefore, a second approach with a thin flexible polyimide substrate has been developed. Figure 5(b) shows that this approach does not need a second cavity due to its flip chip contacting in combination with its small height. A more detailed overview of both sensor integration processes can be found in [21,23,24].

## 4. Experimental

### 4.1. Non-Flexible Design

To evaluate the feasibility of flow separation/reattachment detection by the presented sensing elements, a series of four elements has been integrated on an airfoil. Printed circuit boards have been used for higher adaptability in integration. Those boards have a size of 5 cm × 2.5 cm and can directly be flush-mounted on the airfoil's surface avoiding any steps in flow direction. This gives us the chance to use different flow sensor types with exactly the same measurement setup.

**Figure 6.**
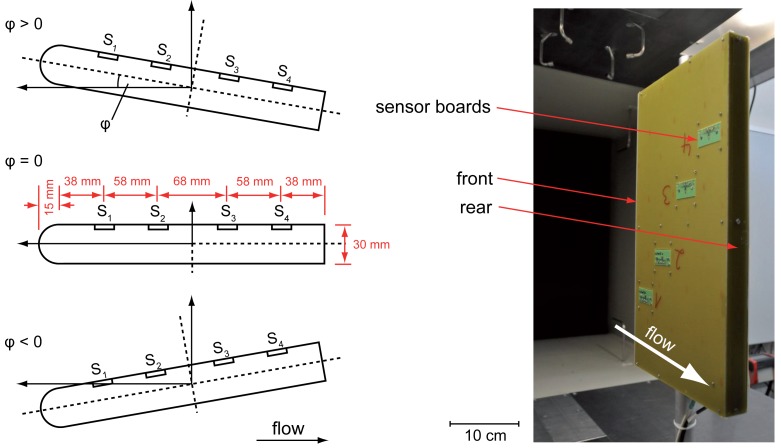
Definition of angle of attack (AOA) (**left**) and (**right**) measurement setup in wind tunnel. The airfoil size is 26cm × 45 cm × 3 cm (length × height × width).

All measurements have been performed in a closed loop wind tunnel at *Deutsche WindGuard Wind Tunnel Services GmbH*. Figure 6 (right) shows the whole measurement setup. As shown in Figure 6 (left), the cross section is directed with the round-shaped side facing the flow to get a preferably low fluidic resistance. A heater power of 15 mW has been chosen, while the whole sensor setup is running in constant voltage mode. The sampling rate is set to 1 kHz with an averaging in a time interval of 1 s. Sampling, measurement and calculations are made with a *National Instruments USB-6212* digital acquisition card combined with *LabView.*

The sensor signals shown in the following are always normalized, meaning a normalization run has been done at defined flow rates to show the existing difference of the thermopile signals for different sensors. Those slight differences are caused by inaccuracies during the sensor fabrication process and cannot be avoided completely. The adaption is small, but for having the right nomenclature, all sensor signals are marked as *mV*(*n*) (normalized) instead of *mV*.

**Figure 7.**
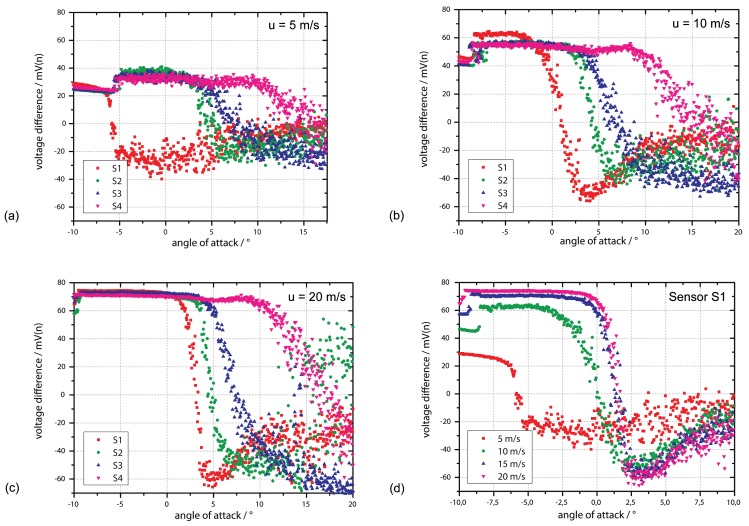
Output signal of the sensors for different angles of attack at 5 m/s (**a**), 10 m/s (**b**) and 20 m/s (**c**) at a heater power of 20 mW. Graph (**d**) shows the output signals of sensor *S*_1_ only. A non-flexible approach is used for the sensors.

In the first measurement, the signals of four equidistantly placed flow sensors (see Figure 6 for exact position) are recorded for constant wind speeds and different angles of attack (*φ,* also pitch) between −10° and +20°. How *φ* is defined can be seen in Figure 6 (right). Figure 7 shows the sensor signals of *S*_1_*, S*_2_, *S*_3_ and *S*_4_ averaged over a time interval of 1 s at wind speeds of 5 m/s (a), 10 m/s (b) and 20 m/s (c). In Figure 7(d) the flow signals of S1 to be compared at different wind speeds are selected only. The resulting graphs show basically the same characteristics for all wind speeds: A constant positive output signal always exists at the starting point with *φ* = −10°. With increasing angle *S*_1_ followed by *S*_2_, *S*_3_ and *S*_4_, the output signals decrease down to zero ending up in negative values with high standard deviation. According to the definitions from Section 3, an output signal of zero shows a vanishing flow velocity in *x*-direction for small values of *y* (close to the wall), a result that could be led back to a flow separation [7,8]. An argument supporting the approach of a pressure-based flow separation is the decreasing output signal, which might occur due to back flow. In contrast, the high noise that can be observed after zero crossing and the sequence of the sensors from *S*_1_ to *S*_4_ do not fit into the scheme. If a flow separation with back flow occurs at *S*_1_, there should definitely be an impact to all following sensors *S*_2_ to *S*_3_. This cannot be observed here, leading to the second possibility of a reattachment of the flow.

**Figure 8.**
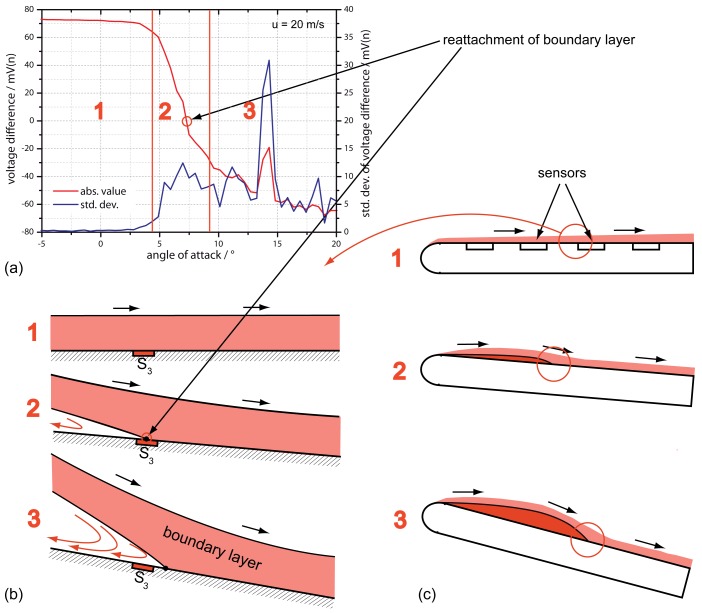
Signal of sensor *S*_3_ at 20 m/s as a function of *φ* (**a**) and its flow conditions (**b,c**) in the areas 1, 2 and 3 at a heater power of 20 mW. The second graph (a) shows the standard deviation of the sensor signal.

To introduce the idea of an observed reattachment of the flow, we should consider Figure 8. Figure 8(a) shows the results of sensor S3 only for different angles of attack as a mean value out of ten seconds as well as the standard deviation of the signal. The output signal shows a clear and constant positive signal in flow direction for *φ* < 3° with a standard deviation of 0.2 mV. With higher angles 3° < *φ* < 7° the flow signal decreases to zero and further down where the standard deviation shows a clear increase. When considering the possibility of a reattachment of the flow passing through, those conditions can be assumed in the same way as shown in Figure 8(b,c) [7,8]. In this assumption, a separation bubble occurs starting at the front of the airfoil, which is rising with increasing angle of attack. With this approach the mentioned problem of the sensor sequence *S*_1_ to *S*_4_ does not occur. Furthermore, the high scattering after zero crossing in Figure 7 and therefore the increase in standard deviation in Figure 8 can be explained: The turbulent conditions increase the integral time scale of the flow, which reduces the independent number of samples acquired.

According to Section 1.2, the phenomenon of an occurring separation bubble expects a *Reynolds* number between 10^4^ and 10^6^[1,7,25]. When working with flow rates between 5 m/s and 20 m/s, the *Reynolds* numbers can be determined using Equation (5) to values between 7.5 × 10^4^ and 3 × 10^5^. The results show that a reattachment due to a transition from laminar to turbulent flow as described in Section 1.2 is possible. Accordingly, the separation itself occurs between the first sensor *S*_1_ and the front of the airfoil. A simple experiment observing this issue is the use of a wool thread showing the direction of the actual flow at each designated position: The experiment indicates a geometric flow separation right at the front of the airfoil forming a separation bubble with backflow close to the airfoil's wall. As measured with the thermal flow sensors, the backflow is characterized by a high standard deviation. After the reattachment point, the wool thread always shows a clear flow direction down to the rear.

**Figure 9.**
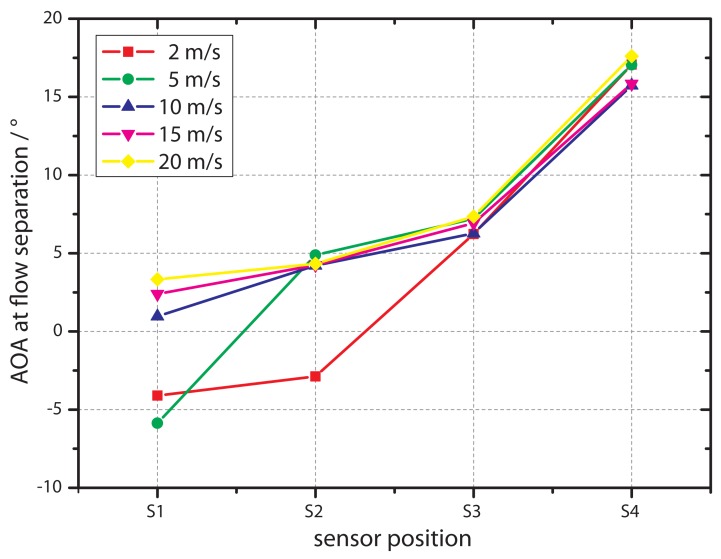
AOA (angle of attack) at a measured flow of zero (by sensor) for different flow velocities in wind tunnel as a function of the sensor position. The graph shows data of non-flexible sensors only.

It is to be mentioned that the behavior of the first sensor *S*_1_ at 2 m/s and 5 m/s, as depicted in Figure 7 and Figure 9, shows a zero flow for a negative angle of attack. There are three possibilities to explain the observed behavior, including the unintended detection of a stagnation point, flow separation point or a reattachment as it occurs on all other sensors. The detection of a stagnation point can be eliminated due to the observed signal trend, which would be negative for higher angles, and vice versa, since the stagnation point has to pass through from rear to front for increasing angles. Here the characteristics are exactly inverted. A detection of a boundary layer separation can also be eliminated, since this would have an effect on the sensor signals of *S*_2_, *S*_3_ and *S*_4_. The most probable reason for the observed sensor behavior of *S*_1_ is a reattachment as well, which also occurs due to a flow separation at the airfoil's front. Flow separation is not expected for this setup, but here it has to be taken into consideration that the used airfoil is not a perfectly flat plate but a structure having a thickness of 30 mm. At lower flow velocities, the flow could have been redirected showing this phenomenon with a reattachment afterwards.

Figure 9 shows the relationship between the angle of attack at zero output and the position of the sensors for flow velocities of 2 m/s, 5 m/s, 10 m/s, 15 m/s and 20 m/s. It illustrates that for the sensors positioned in the back of the airfoil, the AOA at the point of reattachment is equal for all flow velocities. As already mentioned, at *S*_1_ and *S*_2_ differences of the AOA to the sensors in the rear can be observed, especially at lower flow rates of 2 m/s and 5 m/s. Here, even negative values have been measured, giving a further argument that the zero crossing of the AOA is caused by a reattachment. It should be mentioned that the reattachment position at *S*_3_ and *S*_4_ is not sensitive to the flow velocity and, thus, to the Reynolds number. *S*_3_ and *S*_4_ are already close to the rear of the airfoil. Due to that influence, the usual dependency between Reynolds number and length of separation bubble will be different. A lack of accuracy from the data acquisition can be excluded here, since the points of reattachment are easy to identify (low scattering), as shown in Figure 7. A more probable reason is the selected airfoil with its overall length in flow direction of 260 mm and its profile in general.

### 4.2. Flexible Design

Another measurement has been done using thermal flow sensors based on thin flexible substrates. To get a good comparison between both sensor types, the same setup has been used.

**Figure 10.**
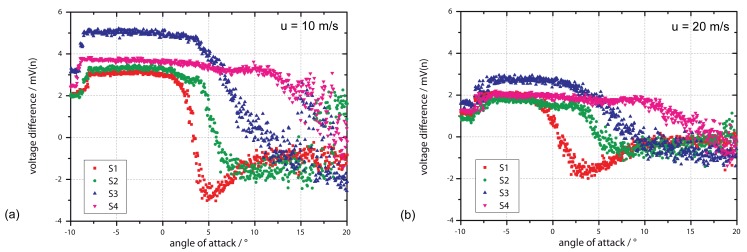
Output signal of the sensors for different angles of attack at 10 m/s (**a**) and 20 m/s (**b**). A flexible approach with a heater power of 20 mW is used here. The high difference of sensor S3 is because a different flexible type has been used at this position.

As shown in Figure 10(a,b), the measured flow characteristics are comparable with the silicon-based sensors. Differences can be seen when considering the absolute signal: here the sensors show a lower reproducibility as well as a lower output signal. The lower reproducibility is derived from the integration process, which has a higher influence on the characteristic line of a flexible approach rather than a non-flexible approach. A lower output signal of the flexible approach comes from the additional polyimide layer as part of the membrane: The polyimide layer increases the heat flux through the membrane, leading to a lower percentage of heat flux going through the fluid. Thus, the sensing element loses sensitivity, but the detection of a flow separation and reattachment is still possible.

## 5. Conclusions

The work presented here is a feasibility study of using thermoelectric-based flow sensors for flow separation and reattachment detection on an airfoil. A non-flexible and a flexible approach based on silicon and polyimide as substrate material has been tested during the work. The integration process shows significant benefits of the flexible approach, which does not need the drilling of a cavity for flush mounting the sensor. However, an integration still needs the effort of an electrical connection, meaning that mechanical work on the airfoil is still required. For measurement, both sensor types have been separately put on an airfoil and tested in a wind tunnel at different wind speeds, angles of attack and on different positions on the surface. A clear zero crossing of the sensor signal could be observed for all settings. Due to further characteristics of the output signals, it could be demonstrated that the observed zero crossing is caused by a reattachment of the flow while the separation itself took place at the front of the airfoil. Hence, it could be proofed by the detection of a reattachment that the sensor setup is basically suited for local detection of flow breakdowns close to the wall and even inverse flow directions. A detection of flow separation could not be demonstrated, but due to the close physical conditions being present at separation and reattachment, a feasibility of the tested sensors for separation detection is expected.

Future work will focus on testing original profiles because the profile tested here with its not curved surface would be not feasible for applications in wind power plants or aviation. Furthermore, the wire-based electrical connections of the flexible flow sensor will be replaced by a wireless energy and data transfer to get a completely non-destructive flow measurement system for airfoils. A first approach on this idea with the flow sensors used in this work has already been presented in [26]. The next step will be an entirely flexible approach with custom electronics [27,28] having a wireless communication and data transfer as well.
